# A specific relationship between musical sophistication and auditory working memory

**DOI:** 10.1038/s41598-022-07568-8

**Published:** 2022-03-03

**Authors:** Meher Lad, Alexander J. Billig, Sukhbinder Kumar, Timothy D. Griffiths

**Affiliations:** 1 Translational and Clinical Research Institute, Newcastle University, Newcastle upon Tyne, UK; 2 Ear Institute, University College London, London, UK; 3 Human Brain Research Laboratory, University of Iowa, Iowa, USA; 4 Biosciences Institute, Newcastle University, Newcastle upon Tyne, UK; 5 Wellcome Centre for Human Neuroimaging, University College London, London, UK

**Keywords:** Auditory system, Learning and memory

## Abstract

Previous studies have found conflicting results between individual measures related to music and fundamental aspects of auditory perception and cognition. The results have been difficult to compare because of different musical measures being used and lack of uniformity in the auditory perceptual and cognitive measures. In this study we used a general construct of musicianship, musical sophistication, that can be applied to populations with widely different backgrounds. We investigated the relationship between musical sophistication and measures of perception and working memory for sound by using a task suitable to measure both. We related scores from the Goldsmiths Musical Sophistication Index to performance on tests of perception and working memory for two acoustic features—frequency and amplitude modulation. The data show that musical sophistication scores are best related to working memory for frequency in an analysis that accounts for age and non-verbal intelligence. Musical sophistication was not significantly associated with working memory for amplitude modulation rate or with the perception of either acoustic feature. The work supports a specific association between musical sophistication and working memory for sound frequency.

## Introduction

Engaging with music (i.e. listening to music, playing an instrument/singing or being involved in any music-related activity) involves processing at multiple sensory, perceptual and cognitive levels. In the pitch domain, it requires an analysis of notes with different sensory properties (frequency structure, temporal envelope and fine-temporal structure) that are associated with different perceptual pitch values. Notes form phrases that are held in mind over short periods of time and compared, a process that requires auditory working memory. The structure of phrases is determined by musical rules that can be learned during musical engagement either implicitly by exposure or explicitly by instruction. Additionally, instrumental performance requires learned sensorimotor coordination.

Musical training, in the form of music lessons, has been linked to advantages in auditory processing and cognitive abilities^1,2^. Participants who have had more music lessons have better melody recognition and ability to predict upcoming melody^3,4^. Musical training can also lead to better fundamental auditory perception. Musicians have been shown to have significantly lower thresholds for frequency discrimination than non-musicians^5^. Classical musicians had an advantage over those trained in contemporary music in pitch discrimination. Additionally, musicians such as flautists who have to adjust the pitch of instruments discriminate finer frequency differences than those playing instruments like piano^6^. However, the effect of musical training, not specific to any instrument or voice training, on frequency discrimination has not been consistently demonstrated^7^.

Musicians may also demonstrate transfer effects from their training to general cognitive abilities such as general intelligence and memory^1,8^. Music lessons in childhood have a positive association with several cognitive abilities in childhood and adulthood. One study showed that students that were randomly assigned music training versus non-musical lessons had a significant increase in their IQ scores after a year^9^. However, cognitive advantages to musicians have not been consistently demonstrated and it may be that these effects are due to people with higher cognitive scores taking or persisting with music lessons or that there are links to preexisting innate interindividual differences^10,11^. There is evidence to suggest that musicians have better memory over shorter time scales than non-musicians and that this effect is moderated by the type of stimuli^12^. Although some studies have shown that musicians outperformed non-musicians in tasks of visual working memory, this has not been consistently demonstrated. There is greater consistency in the demonstration of an advantage of musicians for verbal and tonal stimuli for memory over shorter timescales, than for non-musicians. Auditory working memory may also not be a unitary system and there may be different processes supporting stimuli which can be rehearsed (e.g. words or tonal stimuli) vs those that cannot be (e.g. timbre)^13,14^.

Studies have examined whether the link between music lessons and general cognitive ability are indirectly mediated by specific cognitive domains such as executive function. One study found no significant links between music lessons and tests of working memory (digit span), semantic fluency, conflict management, problem solving and planning^15^. However, another found that splitting executive function into the three different subfunctions of inhibition, updating and switching found significant associations with updating performance and musical ability (these participants also had the highest number of music lessons)^16^. Inhibition here was defined as the ability to control attention, behaviour and thoughts in the face of conflicting information; updating as the ability to continuously monitor information and rapidly add and remove information from working memory; and switching to the ability to flexibly change between tasks or mental sets. The authors concluded that working memory updating abilities might improve via sustained engagement in music. Auditory working memory for tones is an explicit cognitive task that has been examined in a number of studies in which improved performance is shown in groups of musicians compared to non-musicians^12,17,18^. Musicians have also been shown to have improved sustained attention and attention to pitch direction, as well as better general auditory cognition in terms of phonological working memory and speech-in-noise perception^19–22^.

The studies that have tested the relationship between musical instrument experience and perceptual and cognitive abilities have used perceptual and cognitive tasks that differ markedly from each other^19^. There is a need in this area for perceptual and cognitive tasks that are more closely aligned in methodology and output metrics to facilitate the measurement of task effects unique to each domain. For example, perceptual abilities are measured using perceptual thresholds (the amount a stimulus must be changed in order for a stimulus to be perceived as ‘different’) and working memory using a task like the digit span. Although these are standardised tests with a wide body of literature behind them they cannot be used to disentangle the effects of either domain. The digit span also has a numerical verbal component that may act as a confound to performance in the task.

In this study, we consider the extent to which a relationship of any kind with music is associated with improved auditory processing skills: sound perception and working memory. We use the term musical sophistication for this purpose, which has been previously used to capture a wide range of behaviours that relate to music, not necessarily musical training or playing a musical instrument^23^. It can capture the experiences of people from diverse backgrounds such as disc jockeys, ballerinas or music journalists. Musical sophistication is able to capture such ‘engagement’ with music without invoking a requirement of training or playing a musical instrument. The term may also be better suited when participants are studied outside Western society where musical success, excellence and expertise is largely ascribed based on these two domains. Musical sophistication can be measured by using the Goldsmiths Musical Sophistication Index (Gold-MSI)^23^, a validated tool to assess self-reported musical skills and behaviours that captures the variety of musical behaviours in society.

**Figure 1:**
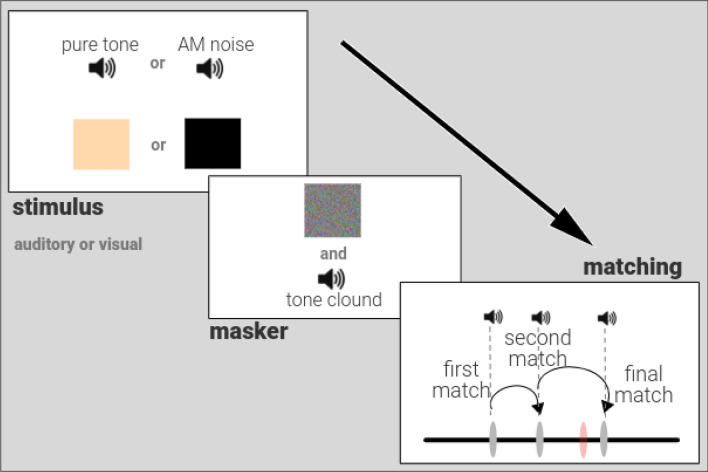
Working memory precision experiment. After an auditory (pure tone or amplitude modulated noise) or a visual (colour or flashing box) stimulus is presented for 1 s, a brief masker (visual and auditory) is presented for 0.5 s. After a subsequent delay of 2–4 s, participants can match to the original stimulus using a horizontal scale on the screen. The scale is linked to the parameter of interest (frequency for pure tone or AM rate) that generates the original stimulus on a given trial and participants can explore the parameter space to ‘find’ the stimulus. The figure shows an auditory matching trial where the participant’s ‘final match’ (dark grey marker on the scale) is shown in comparison to where the original stimulus (orange marker on the scale) is actually located. In this example, the participant first clicked on the scale to make a ‘first match’ (which produced a sound linked to the parameter at that location), then a ‘second match’ and then a ‘final match’. The discrepancy between the ‘final match’ location parameter and that of the original stimulus gives an ‘error’ for each trial that can be used to calculate the auditory working memory ‘precision’, the inverse of the standard deviation of errors from a trial target, for all auditory trials.

We used a novel task that allows a measurement of the effects of perception and working memory via a common output metric. This metric is based on the overall distribution of participant responses across trials in which a sound feature is adjusted to match a target. The adjustable sound is presented either immediately after the target (to test perception) or after a delay and interference (to test working memory). The interference occurs during the matching phase of the working memory task as each attempted match produces a sound, which participants use to decide whether they are further away or close from the sound held in mind. Therefore, with each match, a sound will either have to be updated in their memory store or ignored until a final choice is made (Fig. 1). The matching process is fine-grained to allow a continuous distribution of errors (one for each matching trial) to be calculated. The inverse of the standard deviation of errors then gives the overall metric, termed *precision*, which can be calculated separately for perception and working memory^24^. Previous studies have shown that precision can be successfully used to measure individual working memory resources^25^—here we extend the approach to include perception. We define working memory as the ability to hold non-speech sounds in mind over seconds. The working memory component of this task may also closely resemble the ‘updating’ component described in another study^16^ mentioned above, as it requires the constant monitoring and manipulation of sounds encountered in a trial before a choice is made.

In our study, we examined the relationship between musical sophistication and working memory performance in two domains of sound structure: frequency and amplitude modulation rate, in order to study tonal and atonal components of auditory working memory. Furthermore, we asked whether this relationship is related to perceptual precision for either sound feature. We demonstrate a correlation of musical sophistication with working memory precision for frequency, but not with working memory precision for amplitude modulation rate, nor with perceptual precision for either sound feature. The work defines a specific auditory skill related to musical sophistication.

## Results

### Experiment 1

Experiment 1 examined the relationship between types of working memory and musical sophistication. Figure 1 shows the paradigm to measure auditory working memory precision for tone frequency and for amplitude modulation (AM) rate of sinusoidally modulated noise. It also used a control task based on working memory precision for colour and flash rate of visual stimuli. In both paradigms, subjects had to manually adjust a test stimulus to a target stimulus heard before a delay.

**Figure 2:**
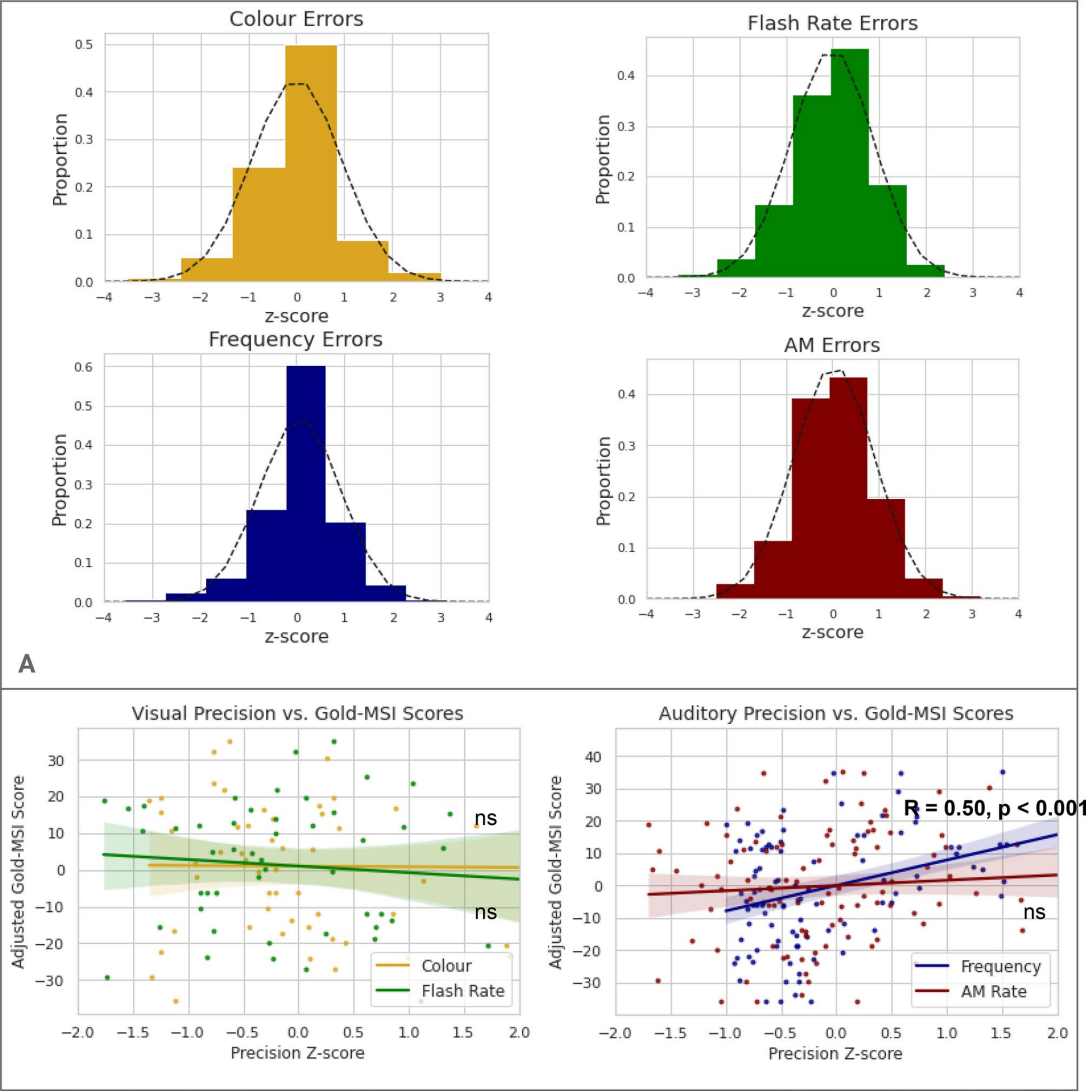
(**A**) The distributions for all domains of working memory tested in Experiment 1 are shown. All participants’ performances were Gaussian. Z-scores are used for errors on the x-axis for the purposes of illustration. (**B**) Scatter plots showing overall working memory precision for visual (top row) and auditory (middle row) stimuli as a function of transformed scores on the Gold-MSI questionnaire. Performance with visual stimuli was not significantly correlated with Gold-MSI scores. For auditory stimuli, working memory precision for frequency (represented by the dark blue line) was significantly correlated with Gold-MSI scores, but working memory precision for AM rate (represented by the red line) was not. *ns* not significant.

Age and non-verbal reasoning scores were used as regressors of no interest for all analyses. Overall, error distributions were Gaussian (Fig. 2A). The range of Gold-MSI scores was 7 to 30 (maximum score is 49) for the ‘musical training’ subsection of the questionnaire (mean 15, standard deviation 7.8). There was a positive correlation between Gold-MSI scores and precision of auditory working memory for frequency, *r*(54) = 0.50, *p* < 0.001 (Fig. 2B). There was no statistically significant correlation between Gold-MSI and precision of auditory working memory for AM rate, *r*(98) = 0.10, *p* = 0.334, visual working memory for colour precision, *r*(50) = − 0.10, *p* = 0.802, or flash rate precision, *r*(50) = − 0.09, *p* = 0.496.

### Experiment 2

**Figure 3:**
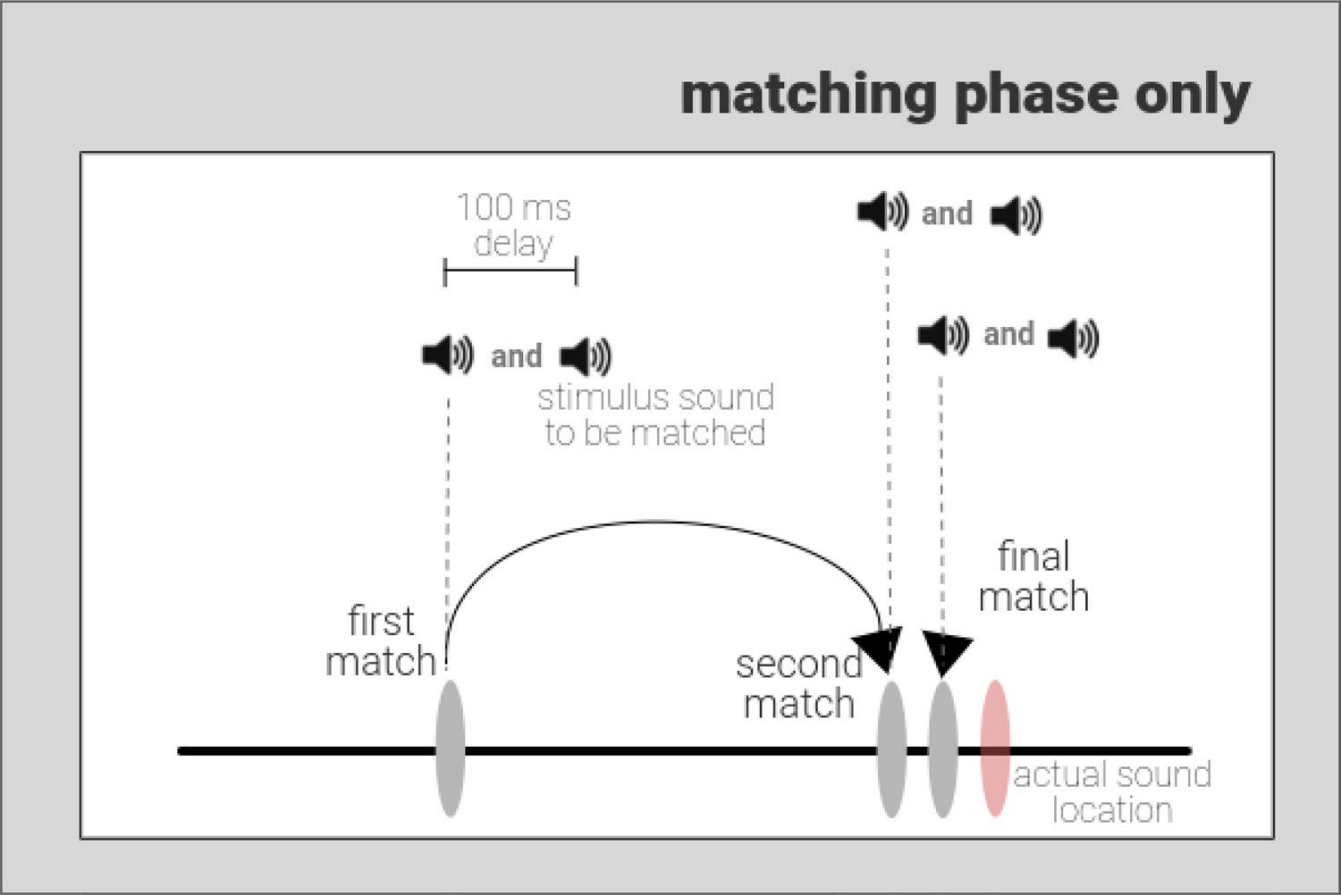
Perceptual precision experiment. In comparison to Experiment 1, there is only a matching phase. The stimulus (auditory trial shown here) to be matched is presented 100 ms after a match is made on the scale and so each click on the scale produces two sounds in a sequence. The first is the sound at the parameter location of the click and the second is the sound to match to. Participants are asked to click multiple times on the scale (hence, ‘first match’, ‘second match’ and ‘final match’) until they find a point where the two sounds in succession sound the same. In this auditory trial, the light red ellipse indicates the true location of the sound parameter to be matched.

After the demonstration of a specific correlation between musical sophistication and working memory for frequency, but not with working memory for amplitude modulation or a visual task, we considered whether there might be a general advantage in frequency analysis that applied to both perception and working memory. Experiment 2 (Fig. 3) measured the precision of auditory working memory for frequency and AM rate in addition to the *perceptual* precision of frequency and AM rate. The aim was to identify whether musical sophistication was associated specifically with the more cognitive task that required a sound to be held in mind for several seconds, or if the association extended to processing of sound over shorter timescales. Additionally, the experiment allowed a further test of the correlation between musical sophistication and auditory working memory for frequency in an independent cohort.

**Figure 4:**
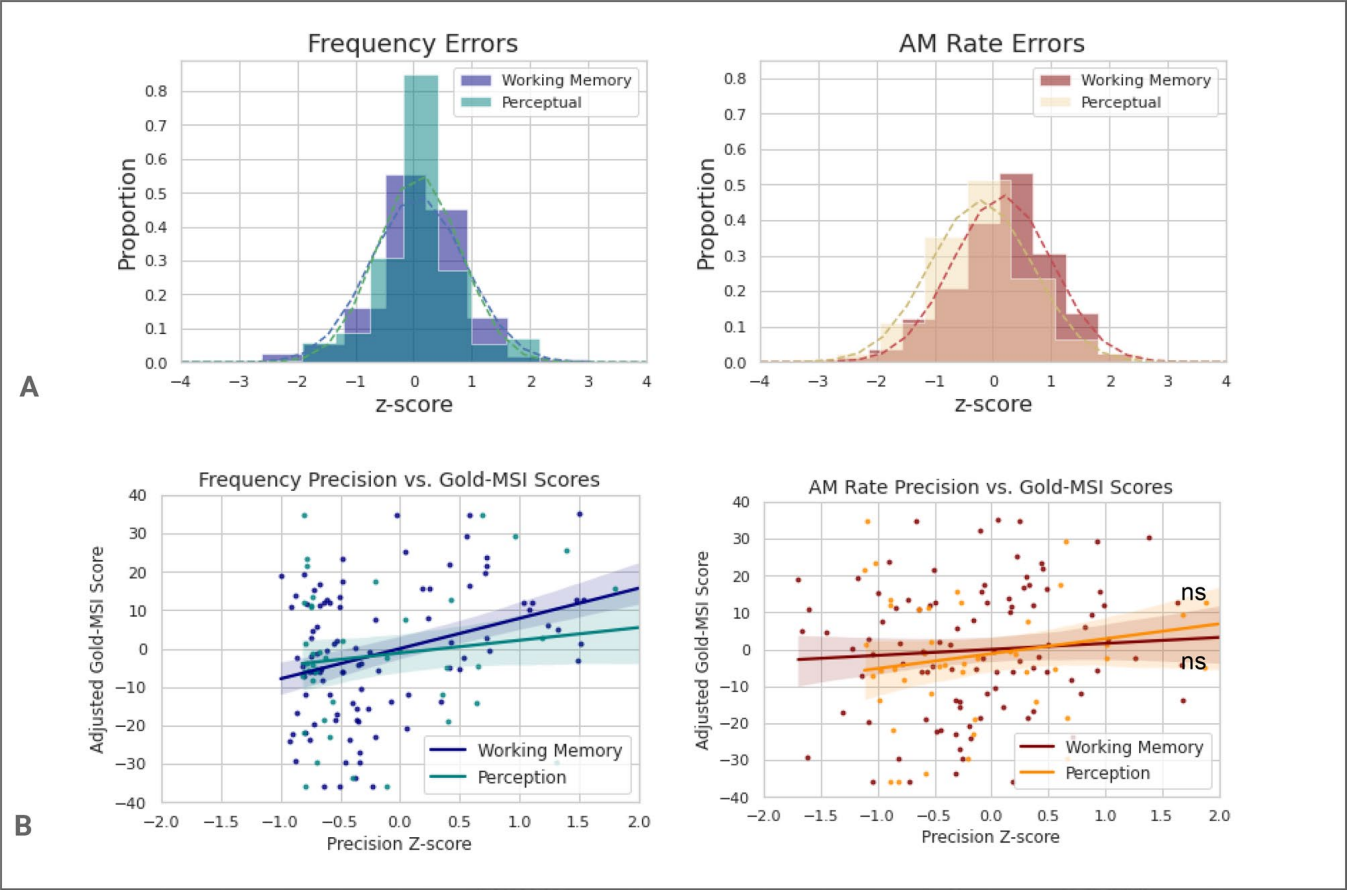
(**A**) The distributions for all auditory domains of working memory and perception tested in Experiment 2 are shown (top row). All participants’ performances were Gaussian but the distribution of perceptual errors has a smaller degree of variability than working memory errors. (**B**) Scatter plots showing overall auditory precision for frequency (left) and amplitude modulation rate (middle row) as a function of scores on the Gold-MSI questionnaire. For tone frequency, precision of working memory (represented by the dark blue line) but not perception (represented by the teal coloured line) was significantly correlated with Gold-MSI scores. For AM rate, neither perceptual nor working memory precision was significantly correlated with Gold-MSI scores. *ns* not significant.

The error distribution for perceptual precision for frequency was narrower than that for working memory (Fig. 4A). The range of Gold-MSI scores was 7 to 28 (maximum score is 49) for the ‘musical training’ subsection of the questionnaire (mean 14, standard deviation 6.7). Neither auditory perceptual precision for frequency, *r*(44) = 0.23, *p* = 0.121, nor perceptual precision for AM rate, *r*(44) = 0.27, *p* = 0.061 correlated significantly with Gold-MSI scores (Fig. 4B). The correlation between the Gold-MSI and precision of working memory for frequency, *r*(48) = 0.42, *p* = 0.003, was significantly greater than that between Gold-MSI and precision of frequency *perception*, *p* < 0.001 (bootstrapped), after permutation testing. Furthermore, the correlation for precision of working memory was significant even after adjusting for frequency perceptual precision (in addition to age and non-verbal reasoning), *r*(43) = 0.37, *p* = 0.009.

## Discussion

This study demonstrates a significant correlation between the precision for frequency working memory and musical sophistication. The correlation is specific to the auditory domain and is not explained by age or non-verbal intelligence. The correlation is present for the precision of frequency working memory but not the precision of AM working memory.

### Musical sophistication is related to frequency but not AM auditory processing

Frequency is a determinant of pitch, a fundamental aspect of music. Changes in pitch form the basis of melody and combinations of pitch form the basis of harmony. These aspects of music are common to many musical cultures and styles. Our study tested participants using a frequency range between 440 and 880 Hz, corresponding to the fundamental frequencies of notes commonly encountered in western music. The range of amplitude modulation rates used in our study (below 20 Hz) is not associated with pitch. It is within the range that tremolo can be added to sung and instrumental notes, for which note-by-note comparison or comparison over phrases is not as critical to the musical experience.

The lack of a relationship between working memory for amplitude modulation rate and musical sophistication may reflect the relative importance of pitch and amplitude modulation in music. We used frequency values that are all within the pitch range and speculate that the result for frequency is related to the importance of pitch in mind in musical culture. Further work is needed to examine more natural pitch-associated stimuli like the harmonic sounds produced by many instruments. Amplitude modulation can be associated with a weak pitch at rates above the lower limit of pitch (about 30 Hz) but we used rates here well below that. Modulation at these rates can give rise to the timbral property or tremolo that is a musical embellishment for which precise rates are rarely required. Our findings are congruent with previous studies showing a particular advantage in working memory for tonal over verbal and visuospatial stimuli in musicians: see^8^ for meta-analysis.

### Musical sophistication is related to working memory precision but not perceptual precision

Previous work suggested that musicians have lower frequency discrimination thresholds than non-musicians^5,19^. Other work suggests that working memory may drive these effects in certain circumstances^26,27^. For example, when the standard is varied on a trial-by-trial basis, working memory might influence performance. Working memory resources help create a ‘perceptual anchor’ when the representations can be easily degraded or interfered with^28^. This is in contrast to fixed standards that are often used to measure frequency perceptual thresholds. In this study we have used a stimulus based on a general precision score for standards that are varied in frequency, per trial, for our perceptual task. However, we do not see any effect of musical sophistication on this perceptual precision. In contrast, we see a clear effect when explicit working-memory demands are imposed based on a longer delay. This dissociation between similar tasks that differ in working memory demands strongly supports a specific relationship between working memory for frequency and musical sophistication.

### Critical aspects of musical sophistication

Musical sophistication may include aural skills, receptive responses, the ability to make or assess musical ability^29^. The Gold-MSI captures this multifaceted nature of musical expertise and is shown to correlate with listening tests of musical ability in the form of melodic memory and musical beat perception^23^. The key elements of the Gold-MSI include five domains which have been derived from factor analysis of a sample of around 150,000 participants. These domains include active engagement, perceptual abilities, musical training, singing abilities and emotional experience. This method is better suited to detect benefits to (or associations with) musicality offered by music communication, journalism or DJing, for example, where instrumental expertise or instruction may not have been attained. Further studies might address an association between specific domains and working memory for frequency using the full version of the Gold-MSI.

### Causal relationship between musical sophistication and auditory processing?

We have demonstrated a specific correlation between musical sophistication and auditory processing that is not explained by a general effect of intelligence. We consider here the nature of possible underlying causal relationships between musical sophistication and auditory processing. The current study, however, cannot provide a definitive answer about these. Previous work has examined relationships between music training and nonmusical abilities and shown that the relationship between these variables is far from direct as implied by correlative study designs^30^.

One assumption is that music training, or musical sophistication in the case of this study, is an environmental effect that induces plasticity in the brain that can then produce transfer gains to other domains. For example, someone who engages in behaviours that lead to a high score for musical sophistication, would over time also increase their abilities in ‘non-musical’ domains like working memory. Our study could then conclude that auditory working memory for frequency rather than visual working memory or working memory for amplitude modulation, or perceptual performance, is ‘enhanced’ by musical sophistication. The counter argument is that people with higher auditory working memory skills would be predisposed to engage in behaviours that increase musical sophistication scores. Our study then would have identified an innate basis for the link between musical sophistication and auditory cognition for frequency. Better innate general listening skills might allow better musical listening and the acquisition of musical sophistication.

At a cognitive level, general natural listening and musical listening both require sustained attention, scene analysis, working memory, rule processing and emotional engagement over several seconds, longer than the sub-second time frame relevant for perception^31^. Alternatively, the additional demands imposed by musical listening in comparison to listening in general could lead to the acquisition of better general listening skills and different behavioural and neural mechanisms for listening. The correlation between auditory working memory and years of musical experience is parsimoniously explained by the acquisition of listening skills^32,33^. Different listening strategies are suggested by functional imaging of musicians carrying out listening tasks that suggest different neural systems for these in musicians^34^.

Other work^11,35^ has described associations with potentially preexisting auditory cognitive abilities that may explain the differences between musicians and nonmusicians. These findings could also be reconciled with our work. Evidence shows that nonmusicians may also have musician-like auditory neurobiological function, in the form of neurophysiological markers, and that preexisting factors may play a large role in links between musical training and cognitive abilities^35^. Although that work^35^ did not measure a musical sophistication index, it could be directly related to our findings as well.

There are several limitations of this study which could be addressed in further work. Firstly, a much larger study with the inclusion of other variables of interest such as the socioeconomic status or participants and parental education may provide more informative links between musical sophistication and auditory working memory, and could also allow us to analyse relationships with the subdomains of the Gold-MSI. This study did not also measure musical ability in any way and relied on self-reported measures of musical sophistication. Although scores on this questionnaire correlate well with tests of musical ability, such as melody or rhythm detection, further research is needed using similar online measures of musical ability such as the Musical Ear Test^23,36^. The nature of the method used to study our participants did not allow us to get detailed information about the computer setup they were using, which may have been very important in certain tasks such as that for perceptual precision. Further work could test these relationships in the laboratory as well as online with new methods to improve the quality of out-of-laboratory auditory measurements^37^. We used a proxy of general intelligence in the form of non-verbal reasoning scores. Further work should include a fuller battery to calculate an individual’s intelligence quotient. However, the finding of a specific relationship between musical sophistication and precision of frequency working memory but not the other precision measures provides some evidence against a third variable (such as socioeconomic status or quality of audio equipment) accounting for our results. Finally, a longitudinal study design with a randomised music enrichment intervention may shed light on direct causal links between musical sophistication and auditory working memory.

## Materials and methods

This study was pre-registered on the Open Science Framework online registry which can be found on https://osf.io/2n58c. Raw tabular data and experimental code is available online linked to the same registry and can be used in line with the CC0 1.0 Universal License. All methods were carried out in accordance with relevant guidelines and regulations. All experimental protocols were approved by the Newcastle University Ethics Committee. Informed consent was obtained from all subjects.

Our previous work^33^, using the same working memory paradigm described in Experiment 1, has indicated that sample size of around 50 is needed to obtain an expected correlation of around 0.40, with years of musical training as a variable, with a two-tailed α of 0.05 and a β of 0.20 and so we aimed to recruit around 50 participants each to each individual study. 102 participants were recruited for two online experiments, designed in the form of a web application, that were created using HTML, CSS and Javascript and hosted freely on Firebase, a platform developed by Google for web developers to create bespoke web applications. The web application prevented participants from performing the task on a mobile device such as an iPad or mobile phone and participants were mandated to wear headphones for the experiments and use a desktop or laptop computer. Participants were paid with £10 shopping vouchers and each experiment took around 25 min to complete.

### Experiment 1

#### Participants

In Experiment 1, 54 participants were identified from a local university database for behavioural experiments. Participants for Experiment 1 were aged 18–80 (mean 32, standard deviation 17).

#### Experimental task

Participants watched a video with instructions to each section of the task and were then able to practice a trial from each stimulus modality of the working memory section once to familiarise themselves with the task. The entire task was divided into 3 sections (working memory task, questions from the Gold-MSI questionnaire and non-verbal reasoning task) which were split further into subsections to reduce the monotony of performing one task over longer periods. Participants had an untimed break after 10 working memory trials, 6 questions or 4 non-verbal reasoning trials. Task order was counterbalanced across participants. Demographic information such as age and sex, and information regarding the set up used while performing the task with regards to the computer (desktop or laptop only) and headphones (in-ear or over-ear only) were obtained after the experiment.

Experiment 1 was designed to test working memory precision in two different visual and auditory domains. 56 trials that consisted of randomly generated stimuli were used. Pilot studies indicated that a stable precision can be estimated for an individual after 10 trials. We cautiously used 14 trials for each condition. For vision, colour hue stimuli from 0 to 300 and the rate of change of a black box to white with a modulation rate between 5 and 20 Hz were chosen. For audition, pure tones from 440 to 880 Hz and white noise modulated with a sine wave (100% depth) between 5 and 20 Hz were chosen.

A schematic diagram of Experiment 1 is shown in Fig. 1. Participants were asked to keep a visual or auditory stimulus in mind and ‘find’ the stimulus on a fixed horizontal scale that they could interact with after a delay. A black cross at the centre of the screen with a white background marked the start of a trial. The initial stimulus was played for 1 s, followed by a visual and auditory masker. The visual masker was designed as a 400 × 200 pixel screen filled with 10 × 10 pixel squares which randomly changed colour at a rate of 60 Hz. This was accompanied by the auditory masker which consisted of randomly generated 50 ms tone pips (from 440 to 880 Hz) for 0.5 s. After a delay of 2 to 4 s (randomly generated from a uniform distribution between these values), participants viewed a 800 px horizontal line with a mouse-movable marker. To mark the beginning of the ‘Matching’ phase, a random probe stimulus (auditory or visual, depending on the trial) was played for 1 s and an inverted red pointer was shown where *this* stimulus was located on the scale. Participants could freely move the marker and click to generate the stimulus at *the clicked* location for 1 s. When they were satisfied that their click matched the original stimulus of interest, they could press the Return key on a keyboard. The parameter space for the stimulus of interest was mapped linearly to the pixel location of the horizontal scale. The extremes (10% most leftward and 10% most rightward) of the scale were not used for mapping as pilot studies indicated that performance is non-Gaussian and skewed when stimuli are matched at these boundaries. We refer to this task as a working memory task rather than a short-term memory task as it requires one to remember a sound in the face of interference from other sounds, during the matching phase, before a match is made on each trial.

Participants completed the general Goldsmiths Musical Sophistication Index (Gold-MSI) questionnaire, containing 18 questions allowing for the scoring of a general musical sophistication factor^23^. It is a self-report inventory that measures differences in skilled musical behaviours in the general or ‘non-specialist’ population. The questionnaire measures different factors associated with musical sophistication such as: active engagement, perceptual abilities, singing abilities and behaviours related to emotional responses to music. The maximum score is 126.

Participants also completed the Matrix Reasoning task from the 3rd Edition of the Wechsler Adult Intelligence Scale^38^. The original task has 30 questions arranged in order of difficulty. In order to increase engagement with the whole task, the last 12 even numbered questions were presented to participants. The first 3 questions were omitted as pilot studies revealed that these were seldom answered incorrectly. The total raw score was out of 12. This task was chosen as it has been commonly used as a proxy for general intelligence in similar studies and used to test associations with musical training or ability^2^. It also minimises the effect of language ability on performance.

### Experiment 2

#### Participants

In Experiment 2, 48 participants were recruited from prolific.com, an online website for identifying participants interested in behavioural experiments. Participants for Experiment 2 were aged 19–56 (mean 33, standard deviation 10).

#### Experimental task

A schematic diagram of Experiment 2 is shown in Fig. 3. In Experiment 2, only pure tone and amplitude modulated auditory stimuli were used. The design of the trials and experimental setup was identical except that in the place of visual trials participants performed the perceptual tasks.

In this experiment, along with the working memory task, participants performed a perceptual matching task interleaved in blocks. The latter began at the ‘Matching’ phase. For every trial participants were played an initial probe sound followed by the stimulus sound, after 100 ms, that needed to be matched. Participants had to make repeated match attempts to find the location on the horizontal scale where the two sounds matched exactly.

### Data analysis

For every working memory trial, an error metric was calculated as the difference between the parameter of the initial stimulus and that which it was matched to. For colour matching trials these errors were used to calculate working memory precision as the inverse of the standard deviation of the distribution of errors across colour trials of the whole experiment. For flash rate, frequency and amplitude modulation matching trials, the error was divided by the parameter of the stimulus to be matched first. This was carried out as for these trials the difference in percept of two images or sounds separated by the same parameter distance reduces as the value of the parameter increases i.e. two sounds pairs with a frequency of 440 and 480, and 840 and 880 are not perceived as similarly different and therefore the first pair has a greater ‘distance’ in musical notation. For Experiment 2, a similar analysis was carried out for auditory perception trials which yielded a metric for perceptual precision. In order to increase interpretability for precision values, these were converted into z-scores for individuals across all the participants.

Descriptive statistics and correlative analysis (Pearson’s Correlation) was carried out using the Pingouin module in Python. Age and Matrix Reasoning scores were used as variables of no interest in subsequent correlative analysis and so the relationship of these variables with Gold-MSI scores were first removed. Residuals from a correlation with age and non-verbal reasoning scores and Gold-MSI scores were calculated first. This allows one to remove partial correlations with both of these variables before carrying out a correlation with our variables of interest such as the working memory and perceptual metrics. For Experiment 2, we performed an additional step of partialling out the effect of perceptual precision for frequency to test whether the relationship between Gold-MSI scores and working memory precision for frequency still remained. Permutation analysis with replacement was used to generate 5000 samples for associations between frequency versus AM rate working memory precision and adjusted Gold-MSI scores, and frequency working memory versus perceptual precision and adjusted Gold-MSI scores. A *t* test was subsequently used to test statistical significance for the difference in these associations. The Holm–Bonferroni method was used to test statistical significance after multiple comparisons.
